# Comparison of an injectable toltrazuril-gleptoferron (Forceris®) and an oral toltrazuril (Baycox®) + injectable iron dextran for the control of experimentally induced piglet cystoisosporosis

**DOI:** 10.1186/s13071-018-2797-5

**Published:** 2018-03-27

**Authors:** Anja Joachim, Aruna Shrestha, Barbara Freudenschuss, Nicola Palmieri, Barbara Hinney, Hamadi Karembe, Daniel Sperling

**Affiliations:** 1 Institute of Parasitology, Department of Pathobiology, University of Veterinary Medicine Vienna, Veterinaerplatz 1, A-1210 Vienna, Austria; 2 CEVA Santé Animale, 10 avenue de la Ballastière, 33500 Libourne, France

**Keywords:** Pig, Swine, Coccidiosis, *Cystoisospora suis*, *Isospora suis*, Diarrhoea, Experimental infection, Toltrazuril, Efficacy

## Abstract

**Background:**

*Cystoisospora suis* causes diarrhoeal disease and reduced weight gain in suckling piglets, and a toltrazuril-based oral suspension is available for treatment. Recently a combinatorial product with toltrazuril plus iron has been developed for parenteral application. In this study we compared the efficacy of the injectable product with the oral suspension against experimentally induced piglet cystoisosporosis.

**Methods:**

In a randomised controlled study, three groups of piglets (*n* = 10–13) were treated either with a fixed dose of 45 mg toltrazuril + 200 mg gleptoferron i.m. per piglet (Forceris®) on the second day of life (study day 2; SD 2) or with 20 mg toltrazuril/kg body weight as an oral suspension (Baycox® 5%) on SD 4 or left untreated (Control group). The Baycox® and the Control group received 200 mg of iron dextran/piglet on SD 2. All piglets were infected with 1000 sporulated *C. suis* oocysts on SD 3. Faecal samples were taken daily from SD 7 to SD 20 to determine faecal consistency, oocyst shedding and other diarrhoeal pathogens. Body weight was recorded on SD 1 and then weekly until SD 29. Animals were observed daily for general health and after treatment for possible adverse events.

**Results:**

In the Control group all animals shed oocysts for 3.1 days on average and all animals showed diarrhoea for an average of five days. Excretion peaked on SD 9 (max. 48,618 oocysts per gram of faeces). Treatment with Forceris® completely suppressed oocyst excretion. In the Baycox® group, low levels of excretion could be detected. Diarrhoea was reduced to single piglets in the treated groups. Body weight development was reduced in the Control group compared to the treated groups. Enteropathogenic bacteria (*Escherichia coli*, *Clostridium perfringens*) could be detected. All parameters related to oocyst excretion, faecal consistency and weight gain were significantly improved in the treated groups compared to the Control group without significant differences between the treated groups. Both products were safe to use.

**Conclusions:**

Treatment with both the injectable (Forceris®) and the oral (Baycox®) formulation of toltrazuril in the prepatent period were safe and highly effective against experimental infection with *C. suis* in newborn piglets.

## Background

Porcine neonatal coccidiosis caused by *Cystoisospora suis* (syn. *Isospora suis*) is a major cause of diarrhoea and unthriftiness in piglets worldwide [1–6] and induces substantial economic losses in the pig breeding industry [7–10]. Currently, the only effective chemotherapeutic drug available for treatment is the triazinetrione toltrazuril [11]. It is available as a 5% suspension to be applied as a single oral treatment during the third to fifth day of life. Oral treatment with toltrazuril is highly effective in controlling oocyst excretion and diarrhoea both in experimental and natural infections of suckling piglets [12–17].

Recently an injectable combination product, toltrazuril + iron (30 mg toltrazuril/ml; 133.4 mg iron/ml as gleptoferron) has been developed for the prevention of piglet coccidiosis together with a prevention of iron deficiency (Forceris®, Ceva, Libourne, France). Treatment is scheduled from the first to the third day of life (i.e. between 24 and 96 h after birth) as a single intramuscular injection of 1.5 ml/piglet corresponding to 45 mg of toltrazuril and 200 mg of iron.

Forceris® is the first toltrazuril product to be marketed in an injectable formulation for the control of *C. suis* in piglets from the first day of life, and this study aimed to evaluate its efficacy against porcine coccidiosis in an experimental infection model [18] in comparison to an established reference product, an oral toltrazuril suspension (Baycox® 5%, Bayer Animal Health, Monheim, Germany).

## Methods

### Animals and husbandry

A total of 35 piglets from four litters were enrolled in the trial on study day (SD) 1 and finished it on SD 29. Sows (Landrace × Large White) were moved to the large animal facilities of the Institute of Parasitology of the Vetmeduni Vienna two weeks before farrowing to acclimatise to the housing conditions. Animals were kept on straw with a heat lamp for the piglets and fed with conventional feed free of coccidiostats. Water was provided *ad libitum*. On the first day of the study (SD 1; within 24 h after birth of the piglets) animals were marked individually and enrolled in the study if they were clinically healthy and weighed at least 900 g. They were randomly allocated to one of three groups according to body weight on SD 1 in the order of the birth of the litters. The general health of sows and their piglets was observed and recorded daily from SD 1 to SD 29.

### Treatment

Animals were treated either with toltrazuril + gleptoferron (investigational product: Forceris®, Ceva, France) at a fixed dose of 1.5 ml/piglet corresponding to 45 mg of toltrazuril and 200 mg of iron, i.m. on SD 2 (Forceris® group, *n* =13 piglets) or with a toltrazuril suspension (reference product: Baycox® 5%, Bayer Animal Health, Monheim, Germany) at a dose of 20 mg/kg orally on SD 4 (Baycox® group, *n* = 12 piglets). A third group (Control group, *n* = 10 piglets) remained untreated. The Baycox® and the Control groups received iron dextran (Uniferon® 200, Virbac, Kolding, Denmark) at a fixed dose of 200 mg/piglet, i.m. on SD 2. Allocation to treatment groups and treatment were carried out under blinded conditions, i.e. only the dispenser was aware of the allocation of piglets to a treatment group during the course of the study. Post-treatment observations for any adverse events (swelling/bleeding of the injection site, inability to stand, walk, suckle or other abnormal behaviour, including dyspnoea, vomiting, limping, lateral recumbency, signs of pain, distress or neurological alterations, [19]) were conducted under blinded conditions by a veterinarian at 2, 6 and 24 h after treatment, and after that daily until SD 8.

### Experimental infection

Each piglet was orally infected with a single dose of approximately 1000 sporulated oocysts of *C. suis*, strain Wien-I [15] on SD 3.

### Determination of concomitant bacterial and viral infections

On the first day of faecal sampling (SD 7) pooled faecal samples from each litter (*n* = 4) were submitted for bacteriological and virological examination to the Institute of Microbiology and Institute of Virology, respectively, of the Vetmeduni Vienna. A general qualitative/semiquantitative bacteriological examination, followed by specific differentiation and virulence factor/toxin typing in positive cases, was conducted. Porcine rotavirus A antigen and nucleic acids of porcine coronaviruses TGEV and PRCoV were determined from the faecal material. In case of diarrhoea after treatment with toltrazuril, individual samples were taken on the first day of diarrhoea and examined for bacterial pathogens as well.

### Efficacy parameters

Individual faecal samples were taken daily from SD 7 to SD 20. Efficacy of toltrazuril was evaluated by qualitative (autofluorescence; [20]) and quantitative (McMaster counting of oocysts per gram of faeces, OpG) determination of oocysts and faecal consistency as previously described [18]. Faecal consistency of each sample was scored immediately with faecal score (FS) 1 describing firm, FS 2 pasty, FS 3 semi-liquid and FS 4 liquid faeces, with FS 3 and FS 4 considered as diarrhoea. Individual body weight was recorded for each enrolled piglet on SD 1, 8, 15, 22 and 29.

To evaluate the efficacy of treatment, oocyst excretion, faecal consistency and body weight development were analysed statistically. This included the area under the curve (AUC) for quantitative oocyst excretion and FS, the number of days with oocyst excretion/diarrhoea, and the number of animals that showed excretion/diarrhoea in each group, as well as the body weight on each weighing day and body weight gain. Data were evaluated for homogeneity of the groups and analysed by means of the Kruskal-Wallis H-test (for continuous parameters) or by means of the Chi-square test (for discrete parameters). Pairwise comparisons were performed either using Fisher’s exact test (excretion/diarrhoea present or not) or Wilcoxon-Mann-Whitney U-test (other parameters). For all tests, *P*-value correction with Bonferroni was applied.

## Results

### Safety

No animal showed treatment-related adverse events that required veterinary intervention. Two animals from the Forceris® group showed a slight temporary swelling at the injection site within the first day of observation after treatment. Two animals from the Control group had to be treated with Ringer’s lactate due to dehydration.

### Qualitative oocyst excretion determined by autofluorescence

All piglets of the Control group and 25% of the piglets in the Baycox® group excreted oocysts detectable by autofluorescence (AF), while none of the animals in the Forceris® group shed oocysts. The mean duration of oocyst excretion was 1.3 days in the Baycox® group and 3.1 days in the Control group (Table 1).The number of days with AF detectable excretion and the number of piglets that excreted AF detectable oocysts were significantly reduced in the Forceris® and Baycox® groups compared to the Control group without statistical difference between the treatment groups (Table 2).

**Table 1:** McMaster countable oocyst excretion, autofluorescence detectable oocyst excretion and diarrhoea values per group

	Forceris®	Baycox®	Control
No. of piglets	13	12	10
No. of samples over all sampling days	181	168	140
McMaster countable oocyst excretion			
No. (%) positive piglets	0 (0)	2 (16.7)	9 (90.0)
No. (%) excretion days	0 (0)	3 (1.8)	21 (15.0)
Autofluorescence detectable oocyst excretion			
No. (%) positive piglets	0 (0)	3 (25.0)	10 (100)
No. (%) excretion days	0 (0)	4 (2.4)	31 (22.1)
Diarrhoea			
No. (%) positive piglets	2 (15.4)	4 (33.3)	10 (100)
No. (%) diarrhoea days	3 (1.7)	6 (3.6)	50 (35.7)
Liquid diarrhoea - faecal score (FS) 4			
No. (%) FS 4 piglets	1 (7.7)	1 (8.3)	7 (70.0)
No. (%) FS 4 days	1 (0.6)	3 (1.8)	22 (15.7)

**Table 2:** *P*-values (given as -log10) are given for the parameters of oocyst excretion and faecal score. Differences at *P* < 0.05 are indicated in bold

Parameter	Forceris® *vs* Control	Baycox® *vs* Control	Forceris® *vs* Baycox	*χ*^2^, *df*
Area under the Curve for OpG	**3.89**	**3.19**	0.35	24.87, 2
No. of days with McMaster countable excretion	**3.89**	**2.70**	0.35	23.17, 2
McMaster countable excretion present or not	**4.43**	**2.24**	0.18	23.09, 2
No. of days with AF detectable excretion	**4.62**	**3.80**	0.70	28.29, 2
AF detectable excretion present or not	**5.52**	**2.85**	0.54	25.36, 2
Area under the Curve for FS	**3.72**	**4.72**	0.00	20.64, 2
No. of days with diarrhoea	**4.13**	**3.60**	0.00	24.46, 2
Diarrhoea present or not	**3.70**	**2.29**	0.00	17.44, 2
Daily body weight gain SD 1–29	**1.56**	**2.59**	0.88	14.59, 2

*Abbreviations*: *OpG *oocysts per gram of faeces, *AF* autofluorescence, *FS* faecal score, *χ*^*2*^ and *df* statistics and degrees of freedom according to Kruskal-Wallis rank sum test or Chi-square test

### Quantitative oocyst excretion determined by McMaster counting

McMaster countable oocyst excretion was observed in nine out of ten piglets of the Control group, in two animals of the Baycox® group and in none of the piglets of the Forceris® group. When excretion days were evaluated, positive animals in the Control group excreted McMaster countable oocysts between one and four days (mean 2.3 days), whereas in the Baycox® group two positive animals excreted for one and two days, respectively (Table 1).

**Fig. 1:**
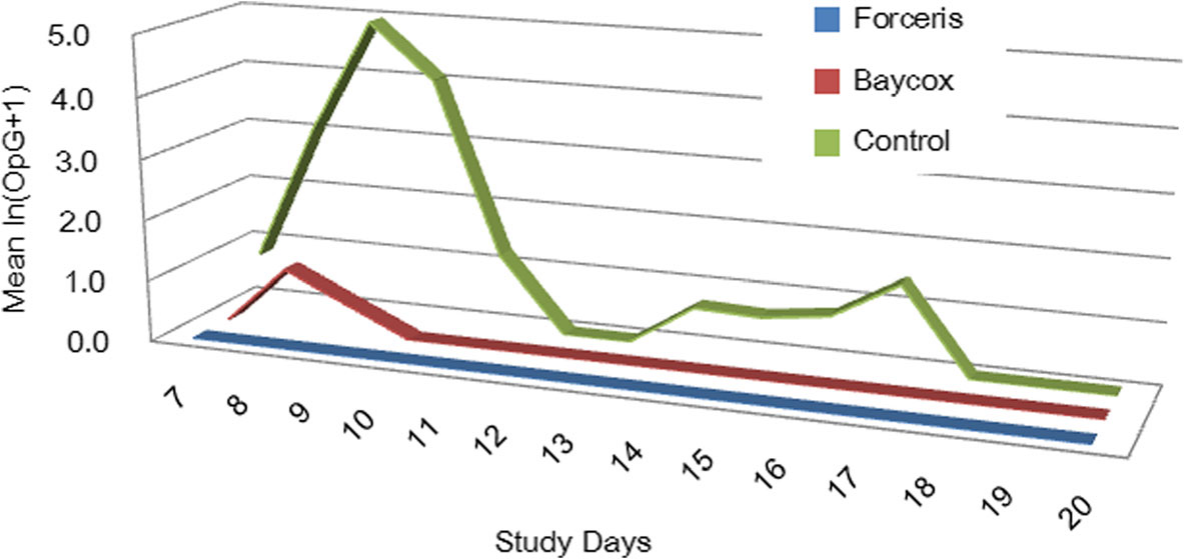
Mean oocyst excretion given as ln (OpG+1) per group during the whole study period

**Fig. 2:**
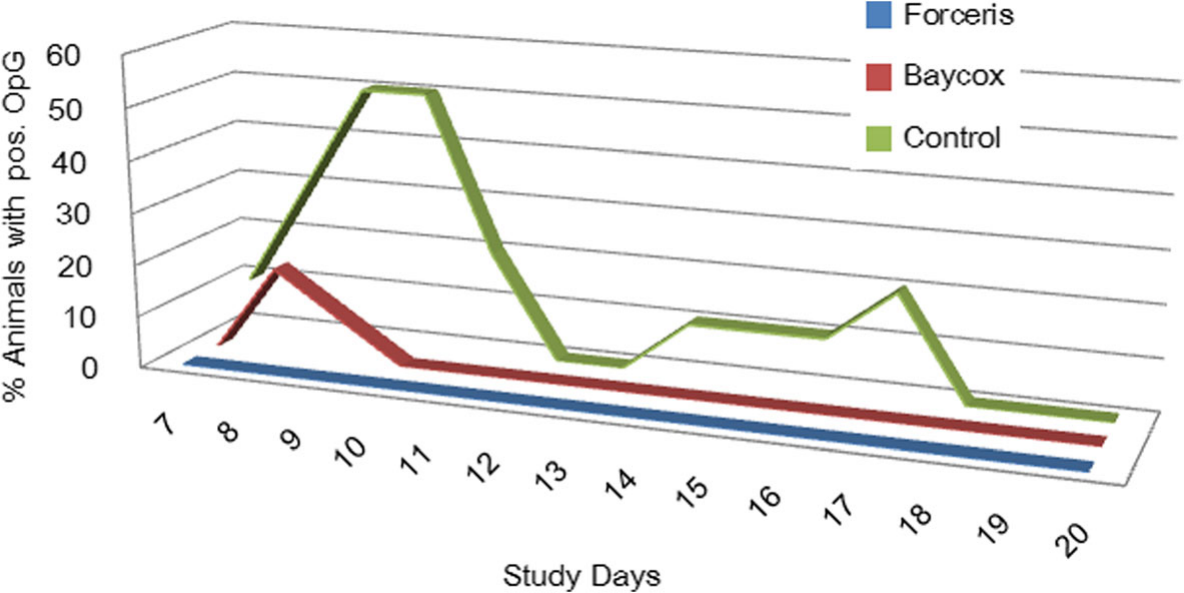
Prevalence of McMaster countable excretion

The maximum OpG value in the Control group was 48,618 OpG on SD 9 (Fig. 1); on that day, the prevalence of McMaster countable excretion also reached its peak at 50% (Fig. 2), while in the Baycox® group the OpGs did not exceed 333.

The area under the curve for OpG, the number of days with McMaster countable excretion and the number of piglets that excreted countable oocyst numbers were significantly reduced in the Forceris® and Baycox® groups compared to the Control group without statistical difference between the treatment groups (Table 2).

### Faecal consistency and diarrhoea

The average faecal score increased above 2 in the Control group from SD 8 to SD 14, while in the treated groups the mean FS never exceeded 2. The maximum prevalence of diarrhoea was 100% in the Control group (SD 11 and SD 12) with an average duration of 5.0 days, while in the Baycox® group 33.3% of the animals showed diarrhoea for an average of 1.5 days, and in the Forceris® group two animals (15.2%) showed diarrhoea for one and two days, respectively. FS 4 (watery diarrhoea) was observed in 70% of the Control animals (average duration: 3.1 days) while in the Baycox® group one animal had watery diarrhoea for three days and in the Forceris® group one animal for one day (Table 1).

**Fig. 3:**
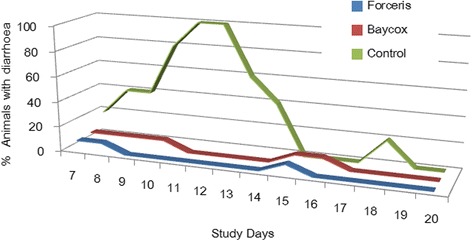
Course of diarrhoea (FS 3 and 4) in the different groups

**Fig. 4:**
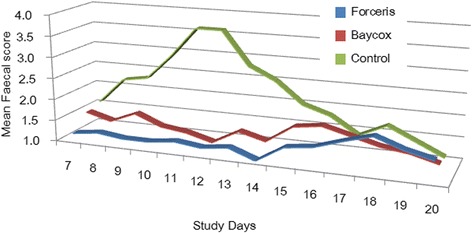
Course of the average faecal score (FS) in the different groups

The peak of diarrhoea was observed on SD 11 to SD 12 when all animals in the Control group showed diarrhoea with a mean FS of 3.6 (Figs. 3 and 4). The area under the curve for the FS, the number of days with diarrhoea and the number of piglets that had diarrhoea were significantly reduced in the Forceris® and Baycox® groups compared to the Control group without statistical difference between the treatment groups (Table 2).

### Body weight development

**Fig. 5:**
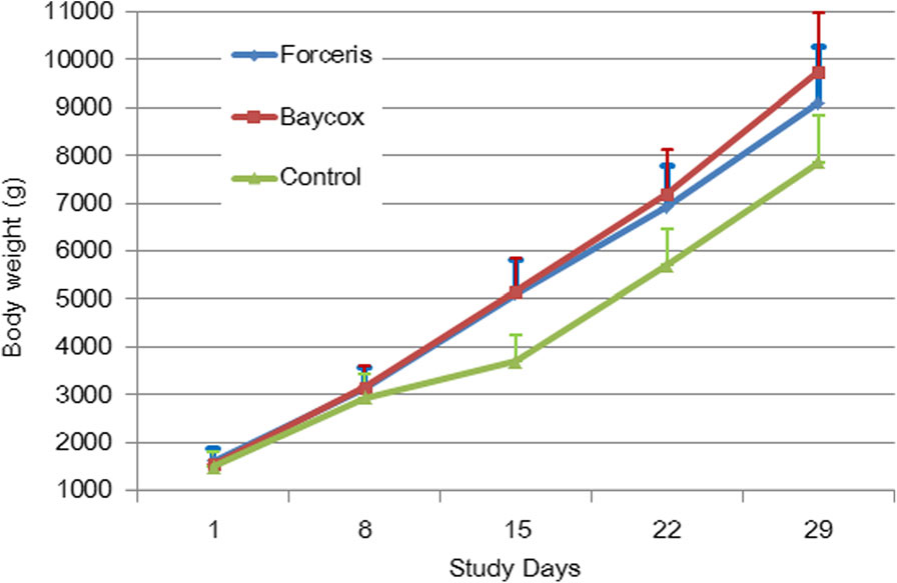
Body weight development during the study (study days 1–29). Vertical lines depict the upper standard deviations

Body weights were not significantly different between the groups on SD 1, the day of randomisation (Kruskal-Wallis H-test, *χ*^2^ = 95145, *df* = 2, *P* = 0.62). Daily body weight gain and total weight gain from SD 1 to SD 29 were lowest in the Control group due to a severe depression of weight gain in the acute phase of infection (from SD 8 to SD 15) during which the Control group only gained 975.8 g on average compared to 2008.3 g in the Baycox® group and 1976.9 g in the Forceris® group (Fig. 5).

The daily body weight gain from SD 1 to SD 29 was significantly higher in the Forceris® and Baycox® groups compared to the Control group without statistical difference between the treatment groups (Fig. 5).

### Concomitant infections

All litters showed infections with a similar pattern of bacteria. Haemolytic *Escherichia coli* which were positive for virulence factors fimH, papC, iucD, and cnf1 and β-2 toxin positive *Clostridium perfringens* Type A were detected in high amounts at the beginning of sampling and were also diagnosed in those animals that showed diarrhoea after treatment. No viral infections were detected.

## Discussion

Piglets experimentally infected with *C. suis* were treated with toltrazuril either parenterally or orally before the onset of oocyst excretion and diarrhoea. Both, Forceris® and Baycox® had a comparable effect on oocyst shedding, faecal consistency and body weight development.

Oocyst shedding was significantly suppressed in both treated groups compared to the Control group. Despite the intra-litter randomisation which entailed a high environmental contamination in all litters due to the presence of untreated control animals, no piglets of the Forceris® group excreted oocysts during the trial. This indicates that the treatment was highly effective and the level of false-positive samples due to coprophagy [21] in such an experimental setting is likely to be low. In the Baycox® group three samples were positive with one oocyst in McMaster counting each, resulting in OpG values of 333. It is difficult to unequivocally conclude that these findings are derived from true infections for the reason stated above; however, one of the two animals that excreted countable oocysts did so on two consecutive days indicating that at least this individual shed oocysts due to infection and not due to coprophagy, but data on the extent of false positive samples due to this in experimentally infected mixed litters are not available. Low levels of oocyst shedding can occur despite treatment with Baycox® [14] while in other experimentally induced infections suppression of oocyst development was complete [15]. Despite low levels of oocyst shedding the reduction of environmental recontamination by disruption of the parasite’s life-cycle was highly effective.

Diarrhoea is a hallmark of porcine cystoisosporosis [2, 3, 18, 22]; it can be induced by parasite infection in the absence of other enteropathogens [23, 24] but exacerbated by rotavirus [24] or *Cl. perfringens* [25, 26], and probably by other enteropathogens as well. Early studies on the use of toltrazuril in pigs indicated that *C. suis* has a forerunner role for bacterial infections as anticoccidial treatment reduced diarrhoea and simultaneously the amount of antibiotics required to control bacterial infections on affected farms, but a low level of diarrhoea was still seen after toltrazuril application [12]. In the light of these findings, it is conceivable that the *E. coli* and clostridia that were circulating in the litters (and also detected in the animals with diarrhoea after treatment) contributed to the clinical outcome in some individuals despite successful treatment of cystoisosporosis.

Body weight development and diarrhoea are inversely related [18] and consequently body weight gain was increased in treated animals (which showed less diarrhoea), with the result of higher weights around weaning on SD 29 compared to the Control group. Improved weight gain has also been shown upon treatment in *C. suis*-positive herds with no apparent clinical signs [27], indicating that the presence of the parasite may influence performance of piglets even at low infection levels.

Parasite shedding, diarrhoea and body weight development showed no significant differences between the two toltrazuril formulations; both were highly efficacious. Forceris® can be used to simultaneously treat metaphylactically against *C. suis* infection and supply the iron required to support the rapid growth and increase in blood volume during the first days of life of the piglets, thus reducing the required handling of the piglets. Time needed for oral treatment of one piglet was estimated at 10 seconds with a labour cost estimation of € 18/hour [27], significantly contributing to the costs of coccidiosis control. Forceris® can be applied from the first day of life (Baycox®: third to fifth day of life) and may thus also be more useful in cases where *C. suis* infections occur very early after birth and are often seriously exacerbated by bacterial infections, such as with *Cl. perfringens* [17]. Accurate dosing of piglets by injection may also be of advantage when vomiting occurs for a variety of reasons, including infection with *C. suis* [28].

## Conclusions

Oocyst shedding was reduced to a minimum in the Baycox® group and suppressed completely in the Forceris® group. Diarrhoea was also reduced significantly in both treatment groups, resulting in significantly better weight gain. The latter was depressed during the acute stage of infection (in the second week of life) in animals that were clinically affected by pasty to watery diarrhoea. Both applications were safe to use and effective in a single application. Early (metaphylactic) treatment of piglet coccidiosis during the prepatent phase of infection can control infection and significantly improve piglet health when *C. suis* is present on a farm. Treatment with toltrazuril together with iron by injection was safe and effective. Handling of animals for medication was reduced by the combination product without interfering with the efficacy of toltrazuril treatment.

## Abbreviations

AF: Autofluorescence
AUC: Area under the curve
FS: Faecal score
OpG: Oocysts per gram of faeces
SD: Study day
